# The Mind From Within: Visceral Roots of Human Cognition

**DOI:** 10.1002/advs.202524184

**Published:** 2026-08-27

**Authors:** Alessandro Monti, Salvatore Maria Aglioti

**Affiliations:** ^1^ Pontifical University of St. Thomas Aquinas (Angelicum) Rome Italy; ^2^ Department of Psychology, Sapienza Università di Roma and Center for Life Nano‐ & Neuro‐Science, Fondazione Istituto Italiano Di Tecnologia (IIT) Rome Italy; ^3^ LIFE Institute for Research and Health Care Santa Lucia IRCCS Rome Italy

**Keywords:** deep embodiment, higher‐order mental functions, homeostasis‐allostasis, multimodal interoception, visceral organs

## Abstract

Humans think and act not only with the brain but also with the heart, lungs, gut, and other viscera, such as the bladder, long regarded as primarily vegetative. A fresh wave of experiments is revealing the role of cardiac‐cycle phase and baroreceptor firing in perception and social behavior; respiration‐locked neural dynamics in attention and learning; and gastrointestinal rhythms and vagal afferents in affect and decision‐making. These discoveries are made possible by emerging methods, including ingestible sensors that record gastrointestinal signals in situ during emotional and cognitive tasks, and virtual reality stimuli linked to cardiac and respiratory activity. Expanding on such evidence, this review advances a deep embodiment theory in which visceral states and their conscious perception (interoception) systematically shape memory, consciousness, volitional action, and social cognition. Deep embodiment has implications for economic decision‐making, clinical interventions (e.g., organ transplantation), environmental exposures (e.g., air quality), and the design of artificial intelligence (AI) systems that incorporate visceral‐like signals for state estimation and adaptive control. Finally, we contrast deep embodiment with brain‐reductionist and “shallow” embodiment accounts, arguing that viscera allow a self‐centered mode of thinking and influence cognition through specific pathways.

## Graphical abstract

The physiological activity of visceral organs, such as the heart, the lungs, and the gut, is surprisingly linked to many sophisticated mental operations, such as remembering the past, being aware of ourselves, making choices, and forging social bonds. This review details the evidence supporting the idea that human mental functions may be deeply embodied.

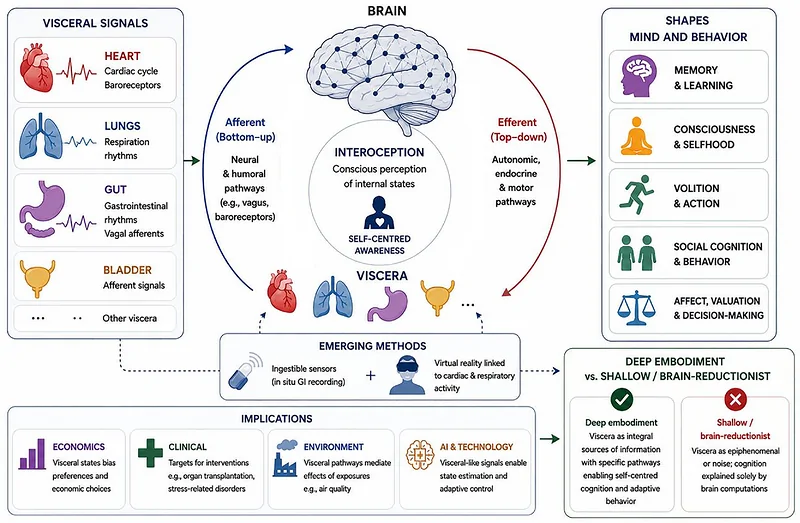

## 1 Introduction

The central nervous system is primed to react not only to the stimuli that come from the external environment, but also to the stimuli that come from the heart, the lungs, the gut, and other visceral organs. A vast array of peripheral receptors is constantly tuned to detect changes in heart rate, breath frequency and amplitude, gastric contractions, and so forth, relying this information to the brainstem and then to the cerebral cortex [1]. In the cardiovascular system, vagal and glossopharyngeal afferents link chemoreceptors and baroreceptors [2] to the solitary nucleus (NTS). The vagus nerve also carries signals from respiratory chemoreceptors and airway stretch receptors to the NTS, which in turn is connected to the thalamus and to the insular cortex. An alternative route connects respiratory nociceptors and cardiac mechanoreceptors to the dorsal root ganglia and then to the thalamus through the dorsal column of the spinal cord [3]. In the gut, epithelial sensors, particularly neuropods [4], relay nutrient and stretch information to the vagus nerve, which in turn projects to the NTS (Figure 1).

**FIGURE 1:**
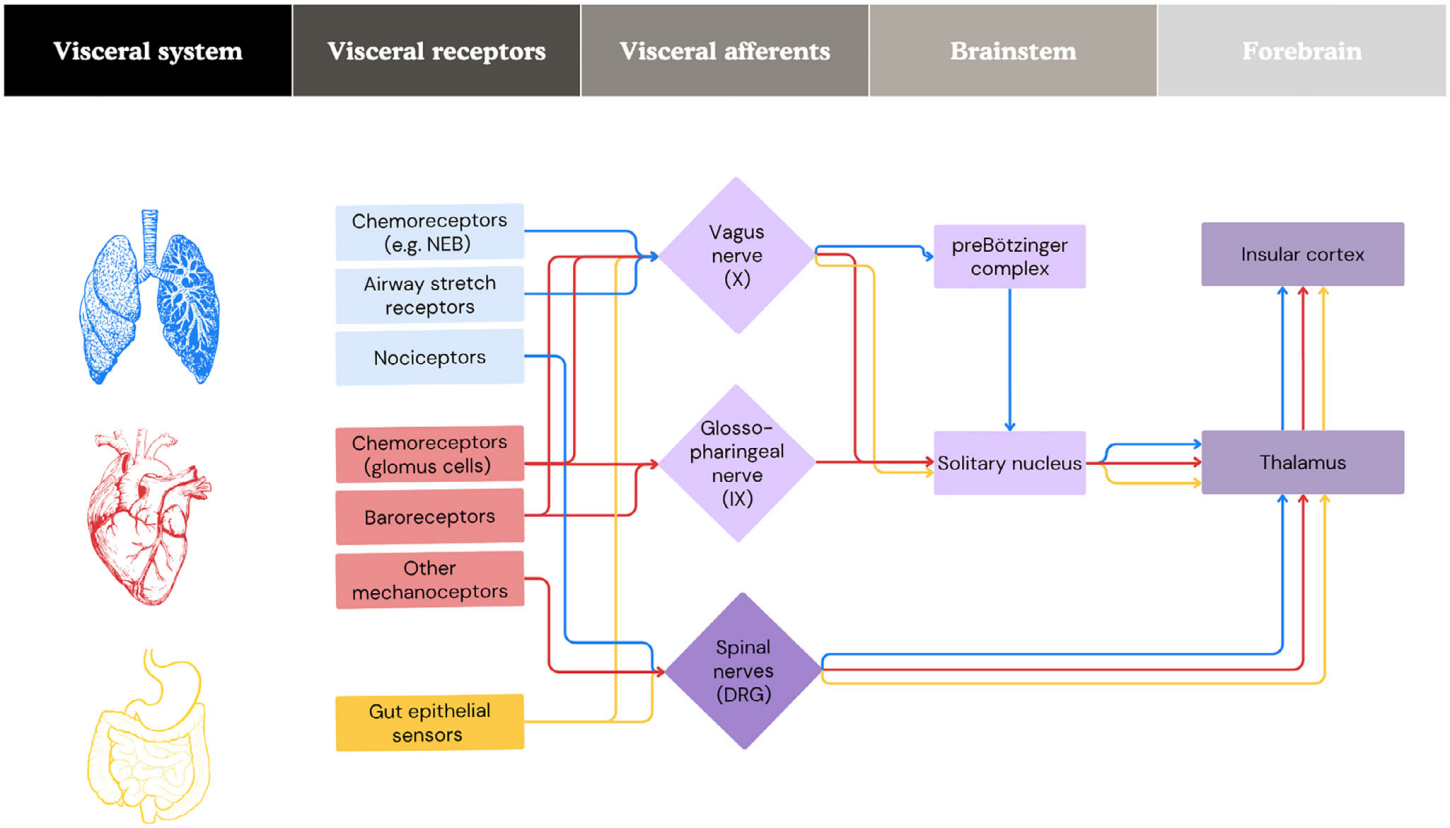
Connections from the visceral organs to the central nervous system. NEB: Neuroepithelial bodies. DRG: Dorsal root ganglia.

Visceral afferents allow the central nervous system to gauge the physiological state of the whole organism and regulate homeostasis. Hence, cognitive neuroscientists could be tempted to regard it as mere noise to be discarded to extract pure brain signals related to mental processes. However, in the last 10 years, several landmark studies have dramatically changed the research landscape, convincingly proving that visceral afferents extend their influence well beyond homeostatic and allostatic reflexes [5]. Indeed, visceral organs and the perception of their activity, known as *interoception*, have a powerful influence on memory, willed action, consciousness, and social cognition—the higher cognitive functions that represent the hallmark of the human species. Yet the impact of this new and promising trend has been partly hampered by the fact that the studies come from disparate, siloed, non‐overlapping scientific fields—fields as diverse as psychophysiology, neuroscience, transplant surgery, public health, and environmental engineering. Hence, it is of paramount importance that experimental techniques originally developed in a research niche become available across research domains, and that key results become integrated in a coherent model.

Visceral signals can be recorded using a variety of methods (Table 1). At the peripheral level, electrocardiography (ECG) gauges heart rate and other cardiac parameters, while electrogastrography (EGG) measures gastric contractions [6]. Furthermore, ingestible pills track parameters as gut acidity, temperature, or intraluminal pressure [7], while wearable breath sensors detect respiratory rate and exhaled gases [8, 9]. At the central level, electroencephalography (EEG) and magnetoencephalography (MEG) allow researchers to find neural signatures of visceral activity, such as heartbeat‐evoked responses (HERs [10], respiratory‐related evoked potentials (RREPs [11] and respiration‐driven brain oscillations [12, 13]. Moreover, functional magnetic resonance imaging (fMRI) highlights neural clusters that are more responsive to visceral cues, like gastric [14] and respiratory [15] resting‐state networks. At the behavioral level, objective tests and self‐reports indicate the extent to which one perceives one's physiological state—an ability known as interoception [16]. It is useful to think of interoception as a three‐pronged construct, consisting of interoceptive accuracy, sensibility, and awareness [17]. *Interoceptive accuracy* is the objective performance on behavioral tests that ask participants to detect heartbeats [18] or other physiological signals, such as breaths [19] or stomach fullness [20]. *Interoceptive sensibility* is the subjective evaluation of how much a participant pays attention to visceral signals, as assessed through questionnaires [21, 22, 23]. Finally, *interoceptive awareness* is the correspondence between accuracy and self‐evaluation of the participant [17].

**TABLE 1:** Main methods in the study of the visceral underpinnings of higher cognitive functions.

Methods in visceral cognition				
Level	Name	Domain	Strengths	Limitations
Peripheral	Electrocardiography (ECG/EKG)	Heart	Temporal resolution Ease of use Standardized analysis	Spatial resolution
	Electrogastrography (EGG)	Stomach	Ease of use	Spatial resolution
	Ingestible pills	Stomach Gut	Wireless technology Multi‐domain sampling	Temporal resolution Cost
	Breathing sensors	Airways	Wearability Temporal resolution	Specificity
Central	Electroencephalography (EEG)	Brain	Temporal resolution Ease of use Standardized analysis	Spatial resolution
	Magnetoencephalography (MEG)	Brain	Spatial resolution Temporal resolution	High cost Large‐size apparatus Low sensitivity to deep areas
	Functional Magnetic Resonance Imaging (fMRI)	Brain	Spatial resolution	Temporal resolution Cost
Behavioral	Objective tests	Interoceptive accuracy	Standardized analysis Causality (w/ lesions) Cost	Construct validity
	Self‐reports	Interoceptive sensibility & awareness	Ease of use Cost	Content validity

The diverse influence of visceral organs on higher cognition can be summarized in three main pathways. As we will see, a *first‐order influence* occurs when visceral activity shapes specific cognitive functions by tuning neural activity. A *second‐order influence* takes place if a visceral organ impacts on another organ, which, in turn, affects cognition. A *third‐order influence* happens when the visceral activity affects the general state of the brain or of the body, and such state facilitates or hinders cognitive operations. Across these three pathways, better interoception generally boosts cognition and promotes a more ‘self‐centered’ mode of thinking. In this review, we will describe the cognitive role of the cardiovascular (section 2), respiratory (section 3), and gastrointestinal system (section 4), together with other, currently underexplored visceral organs (section 5). Then, we will outline how this large and diverse corpus of studies can be interpreted in a unified framework, that we call “deep embodiment” (section 6). We will compare and contrast deep embodiment with other psychological accounts of the relationship between mind and body, that is, brain reductionism and “shallow” embodiment. Finally, we will discuss open issues and suggest new directions for future research, from advanced tools to record deep body activity in real time to artificial intelligence (AI) software that incorporates visceral information.

## 2 Cardiac Underpinnings of Cognition and Emotion

### 2.1 Heart and Memory

If you know something by heart, you remember it very well. Far from being a mere idiom, this expression highlights the role that cardiac signals can exert in encoding and retrieving memories. For example, it has been hypothesized that afferent baroreceptor cues time‐locked to systole target and modulate noradrenergic pathways involved in memory processes [24]. This could explain why participants who had lower interoceptive accuracy, meaning that they had a poorer estimate of their number of heartbeats in a given time window, were also worse at recalling words that were originally presented at systole than at diastole. Those with higher interoceptive accuracy were less susceptible to this systolic interference on memory [24]. In a subsequent experiment, higher interoceptive accuracy, systole‐dependent feedback, and cardiovascular arousal were linked to better learning and memory of fearful stimuli [25]. Fear memories encoded at systole elicit stronger skin conductance responses, and conditioned fear is better induced when both the acquisition and the extinction phases occur at the same point of the cardiac cycle (systole or diastole) [26]. In a similar vein, heart rate and skin conductance time series recorded while undergoing a virtual threat correlate with how much participants later recall they had been aroused during the virtual experience, suggesting that emotional memories reflect physiological patterns of arousal at encoding [27]. Perceiving afferent autonomic body signals could also trigger a feeling of familiarity with a stimulus, and thus improve prospective memory. Indeed, higher cardiac accuracy is also linked with better prospective memory [28]. Shared neural substrates, like the hippocampus, can mediate the interplay between interoception and memory, as shown by the fact that hippocampal‐dependent logical memory and verbal learning improve as a function of cardiac accuracy [29].

### 2.2 Heart, Consciousness, and the Self

Cardiac signals also play a role in consciousness and self‐consciousness, as they accompany or facilitate the conscious perception of external stimuli, the processing of self‐related information, and the embodiment of a body part, or even a whole body (for theoretical perspectives, see [30, 31, 32]). In particular, spontaneous neural activity time‐locked to heartbeats possibly provides the brain with self‐related information contributing to visual subjective experiences [33]. For example, heartbeat‐evoked responses (HERs) preceding the onset of a faint visual grating predicted whether experimental subjects consciously saw the stimulus [34]. In another study, when participants were able to consciously perceive near‐threshold visual stimuli preceded by an alerting tone, their heart rate displayed a more pronounced acceleration‐deceleration pattern than when they missed the stimuli [35]. While cardiac activity preceding or following stimulus onset is linked to improved conscious vision, stimuli presented in sync with heartbeats are less easily detected than those presented out of sync, and induce a weaker activation of the insular cortex [36]. This cardio‐visual suppression effect disappears after lesions of the anterior part of the insula, implying that anterior insular activity mediates cardiac influence on visual awareness [37]. Cardiac signals can even help detect residual awareness in patients suffering from disorders of consciousness [38, 39]. Phase‐shift of the cardiac cycle, heart‐rate variability, and heartbeat‐evoked responses are promising markers of awareness even in absence of overt behavior, as they help distinguish individuals having a minimally conscious state (MCS) from those in a vegetative state/unresponsive wakefulness syndrome (VS/UWS) [40, 41, 42].

Beyond simple awareness, mounting evidence suggests that brain activity time‐locked to heartbeats is involved in self‐consciousness, too. In contrast with the fleeting nature and extreme variability of visual, tactile, and other exteroceptive cues, cardiac signals are relatively steady across the lifespan and thus may anchor subjective experiences to a self‐referential frame [32]. In a landmark experiment, the likelihood that participants spontaneously focused on themselves as subjects (“I”) or objects (“me”) in their thoughts covaried with HERs originating from the ventral precuneus and from the vmPFC, respectively [43]. A follow‐up study using intracranial electroencephalography (iEEG) and functional magnetic resonance imaging (fMRI) further substantiated these conclusions and found a region‐of‐interest (ROI) correlation between “I”‐related thoughts and HERs stemming from the right anterior insula [44]. At the same time, a specific kind of HERs, namely, heartbeat‐evoked potentials (HEPs) recorded through EEG, covary with illusory feelings of embodying a virtual avatar. On average, the higher the amplitude of posterior cingulate cortex HEPs, the more likely participants were to identify with a virtual avatar that was touched in sync with their real body, and to feel the touch on the avatar [45]. Furthermore, hearing one's own name, as opposed to an unknown name, induces different HERs in the temporo‐parietal junction, and pre‐stimulus HERs predict whether participants will hear their own name embedded in white noise [46].

A parallel line of research sought to gauge the impact of heartbeats on bodily self‐consciousness through several forms of ‘cardio‐visual synchrony’, that is, pairing each heartbeat with a flash of light on a virtual rubber hand [47], on the picture of a face [48, 49, 50], or on a full virtual body [51]. The cardio‐visual manipulation of a virtual body is reflected in the activity of the right insular cortex [52] (cf.), bilateral Rolandic operculum [54] (cf. [53]), and parietal somatosensory cortex [55]. Generally speaking, when images of bodies or body parts flash in sync with their heartbeats, participants are more prone to feel them as part of themselves than when they flash out of sync (but see [48]). Such cardio‐visual effects tend to be stronger for individuals with higher interoceptive accuracy, while people with low interoceptive metacognition appear to be less likely to locate their self within their body [56]. Besides heartbeat timing, there is initial evidence that other cardiac parameters may contribute to self‐related cognition. In particular, healthy individuals are able to recognize the sound of their own heart, as recorded through a Doppler device, and distinguish it from the sound of someone else's heart [57].

Poor processing of cardiac signals seems to be linked also to disorders of self‐consciousness like depersonalization‐derealization [58, 59] (but see [60]) and eating disorders [61]. In turn, enhanced attention toward oneself increases performance in the heartbeat detection task [62, 63], at least in Western samples [64], suggesting a closed loop between cardiac interoception and bodily self‐consciousness.

### 2.3 Heart, Willed Action, and Decision Making

Those who “follow their heart” are often thought of as impulsive, even irrational decision‐makers who are scarcely in control of their actions. Nevertheless, recent evidence points out that interoceptive accuracy is positively, if indirectly, correlated with the sense of agency—the feeling that one is controlling one's actions and thus influencing events in the external world [27, 28, 29, 65, 66]. By reducing internal sensory noise, cardiac signals could make it easier to precisely select and control actions, leading to an increased sense of agency [67]. Individuals who count their heartbeats more accurately display a stronger intentional binding, the compression of perceived time between an action and its outcome that can be taken as an implicit measure of the sense of agency [65]. In these individuals, intentional binding is further enhanced during cardiac systole, when interoceptive signaling is stronger, as compared to diastole [67] (cf. [68]).

In contrast with implicit measures of agency, explicit measures don't seem to be affected by cardiac fluctuations [69]. However, when afferent heart signals are stronger and more frequent, as in the systolic phase of the cardiac cycle, bodily and non‐bodily cues are more clearly distinguishable from each other [70]. This may help people with high interoception act more efficiently and less impulsively, as they display a lower degree of non‐planning impulsiveness [70, 71] and are less prone to risky behavior affecting their body [72] (see also [71]). The systolic increase in interoceptive signaling is also associated with better response inhibition on the stop signal task [73] (but see [74]), decreased willingness to start a voluntary movement [75], and slightly faster responses in a cognitive control task [74]. Conversely, those with poor interoception exhibit a more impulsive behavior—as if they had an urge to move the body [76]. Of note, urges to perform involuntary repetitive actions like tics are a hallmark of Tourette's syndrome. There are hints that Tourette's patients may be less accurate than healthy controls in tracking their heartbeats. At the same time, their premonitory urges to tic are positively correlated to their levels of interoception, suggesting that they have heightened, but noisier bodily sensations [77, 78].

Cardiac signals can also shape subtler, more meditated, or more complex decisions. The fact that baroreceptor cues are stronger at systole, and facilitate the processing of threat‐related information, is consistent with the discovery that the cardiac cycle shapes error monitoring and adjustment during a skilled motor action like playing music: pitch errors made at systole yield greater event‐related potentials (ERPs) and have a less pronounced impact on musical performance than those at diastole, while heart rate changes anticipate the occurrence of errors [79]. The heart can play a role even in sophisticated economic decision‐making, as shown by testing individual levels of interoceptive accuracy in a group of high frequency financial traders. On average, traders were better at counting their heartbeats than matched controls. The more accurate a trader was, the more he earned profits and the more likely he was to survive in the financial market [80]. Again, this points to the fact that risks and threats are better dealt with in a state of interoceptive enhancement. The contrary phenomenon, known as ‘interoceptive numbing’, may reduce the perception of threat and increase extremely risky behavior. As a striking example, suicide attempters are worse than controls in a heartbeat tapping task, while their insular cortex—a key interoceptive hub—is less active when they have to focus on sensations coming from their heart [81]. Finally, complex decisions often entail a degree of self‐reflection, which in turn is related to heartbeat‐evoked responses (HERs: see above, section 2.2). As a case in point, HERs are linked to subjective cultural preferences: when healthy participants made pairwise comparisons between popular movies, choosing their favorite movie elicited larger HERs in their ventromedial prefrontal cortex (vmPFC) and post‐central gyrus than deciding which of the two movie titles had the higher visual contrast. The larger the HERs recorded in right anterior vmPFC before option presentation, the greater the activity in right posterior vmPFC during choice, that is, the period in which subjective preference values are encoded. The magnitude of the HER‐vmPFC interaction also predicted the degree to which participants were precise and consistent in their choices [82].

### 2.4 Heart and Social Cognition

Heart‐to‐heart talks are synonymous with frank, sincere discussions in which friends and close relatives reveal their most intimate feelings and thoughts. Many recent experiments are revealing how cardiac activity reflects, and possibly influences, social cognition—one's ability to infer what others think, who they are, and how one should deal with them. Perspective taking and interoception are intertwined at a behavioral as well as physiological level, particularly when adopting an external point of view requires to deal with bodies and emotions [83]. In such contexts, the role of cardiac signals in disentangling what belongs to the self from what is outside of it may prove particularly useful. For example, imagining a friend rather than oneself elicited a different pattern of HERs in the bilateral precuneus and posterior cingulate cortex [84]. In a similar vein, being prompted to rate the emotions of someone else before an affective scene (thus adopting another person's point of view) induced different HERs in the visual cortices and right frontal operculum than being prompted to rate one's own emotions [85]. Another study [86] found that cardio‐visual synchrony in immersive virtual reality makes it easier to imagine taking the perspective of a virtual body, provided that participants display a high empathic ability. People who are worse at perceiving their heartbeats seem to display a more blurred self/other distinction [87] and are more prone to experience their body as an object whose value depends on how it looks to other people [88], although it is unclear whether they are more amenable to the rubber hand illusion than an average sample [47, 89, 90, 91, 92].

Cardiac signals matter also when people interact with each other in an actual or simulated environment and classify individuals according to their racial or social profile. At systole, when cardiac afferent activity and threat perception are enhanced, a sample of White participants was more prone to misidentify tools as weapons after being primed with the picture of a Black face, and weapons as tools after being primed with the picture of a White face. Furthermore, in a shooting simulation, they were more likely to shoot at unarmed Black targets as if they were armed, but not so at diastole [93]. Likewise, pictures of faces presented in sync with one's systole are deemed less trustworthy than those presented out of sync, whereas such a cardiac modulation of perceived trustworthiness does not hold at diastole [94]. Evidence also indicates that at systole those with better interoception display an increased tendency to use their own emotional state when relating to others (emotional egocentricity bias) [95]. On a related note, listening to the sound of one's own heart during the ultimatum game increases not only subjective ratings of unfairness in response to unfair offers, but also the chance of *making* unfair offers [96], while people with high cardiac interoception are willing to tell self‐serving lies even when their reputation is at stake [97]. What is more, social pain is reduced at systole compared to diastole, like physical pain [98]. All in all, this lends further credit to the idea that cardiac afferent signals make people more self‐centered and self‐concerned. Interestingly, in an American sample there was a positive correlation between higher cardiac interoception and political liberalism [99].

While these studies highlighted the importance of cardiac signals for social cognition, a few experiments proved that the reverse is also true. In particular, speech anticipation seems to increase interoceptive accuracy [100, 101] (but see [102]), an effect correlated with fear of negative evaluations [100]. On the contrary, social exclusion makes people less accurate in perceiving their heartbeats, possibly reflecting a defense mechanism that shifts attention away from distressing self‐awareness [103]. Furthermore, adolescents who expect to be rejected in a social rejection task whose outcome is uncertain display more negative heartbeat‐evoked potentials (HEPs) in anterior cingulate, prefrontal, and insular cortices [104]. Such a two‐way relationship between interoception and social cognition might lead to positive or negative feedback loops that either promote or discourage specific forms of social behavior.

### 2.5 Heart Diseases and Higher‐Order Mental Functions

Cardiovascular issues such as heart failure (HF) and transplant can significantly change the manifold relationship between the heart and higher cognitive functions. Some form of cognitive impairment occurs in up to 85% of patients with HF, who have up to a twofold risk to develop memory and executive function deficits compared to healthy controls of their same age [105]. This may be due to the fact that cardiac insufficiency reduces cerebral blood perfusion or leads to cerebral emboli [105] (see also [106]). Such effects need to be controlled for HF risk factors that have an impact on cognition and thus may represent confounding variables, like hypertension, depression, diabetes, and smoking [105].

Although heart transplants may attenuate some of these symptoms, almost 40% of long‐term survivors of heart transplantation have a poor performance on cognitive tests and around 30% qualify for mild cognitive impairment [107]. Of note, interoceptive accuracy decreases for some months after heart transplant, and post‐transplant HEPs exhibit a reduced amplitude even a year later [108]. A fascinating case study [109] tested interoception and social cognition in a patient who ‘feels two hearts’, as he carries an artificial pump to support his failing left ventricle. The patient relied on the somatosensory feelings evoked by such an artificial pump more than on his heart to perform a heartbeat tapping task. Contrary to healthy controls, shifting his attention from exteroceptive to interoceptive feelings did not change his heartbeat‐evoked potentials (HEPs). What is more, his theory of mind was markedly impaired, and he failed to recognize accidental (as opposed to intentional) pain in an empathy for pain task. This clinical evidence further shows the importance of an unimpaired heart‐brain axis for implementing higher cognitive functions in everyday life (Figure 2).

**FIGURE 2:**
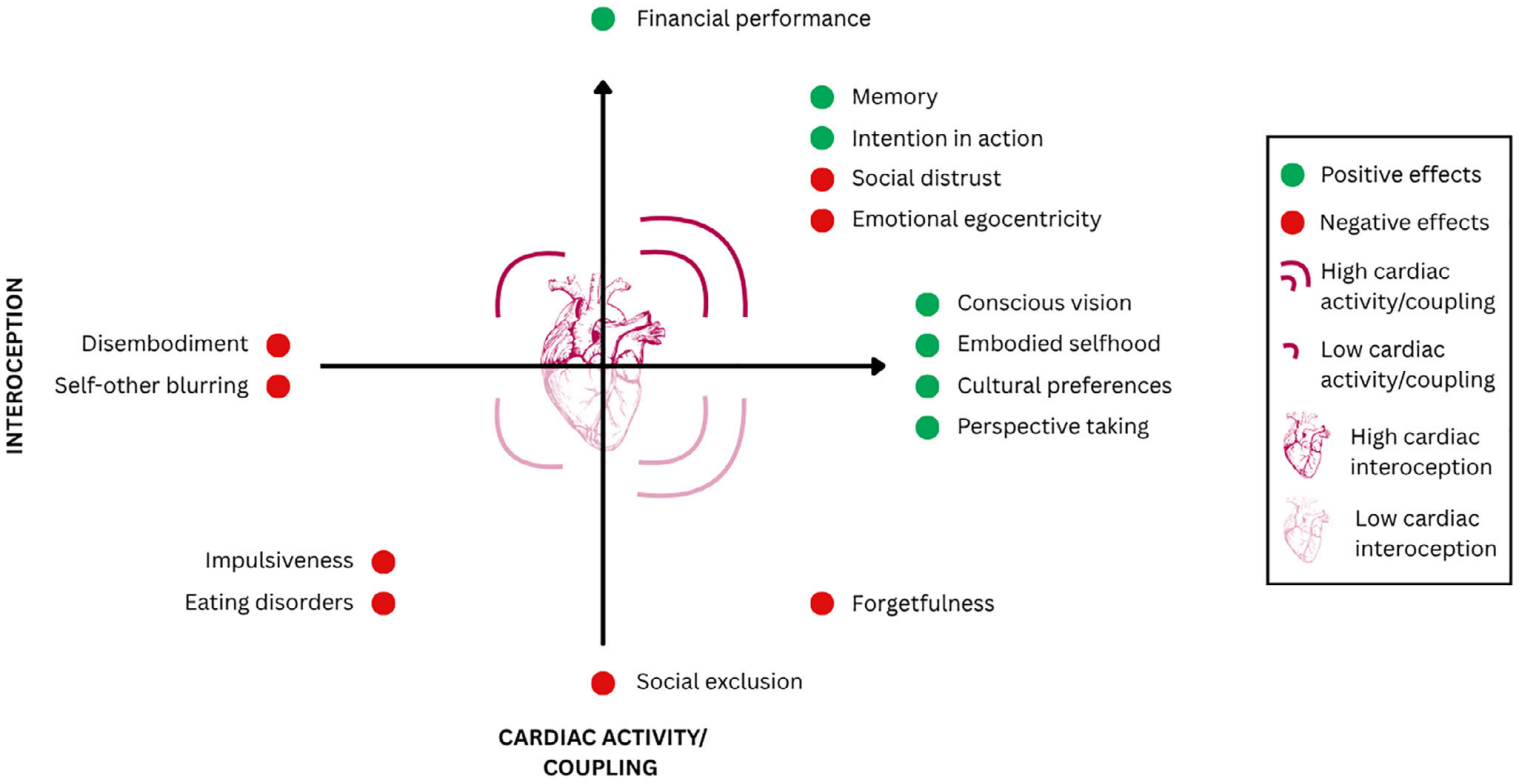
Impact of the cardiovascular system on higher cognitive functions, including the influence of cardiac signals coupled with brain or visual signals.

**FIGURE 3:**
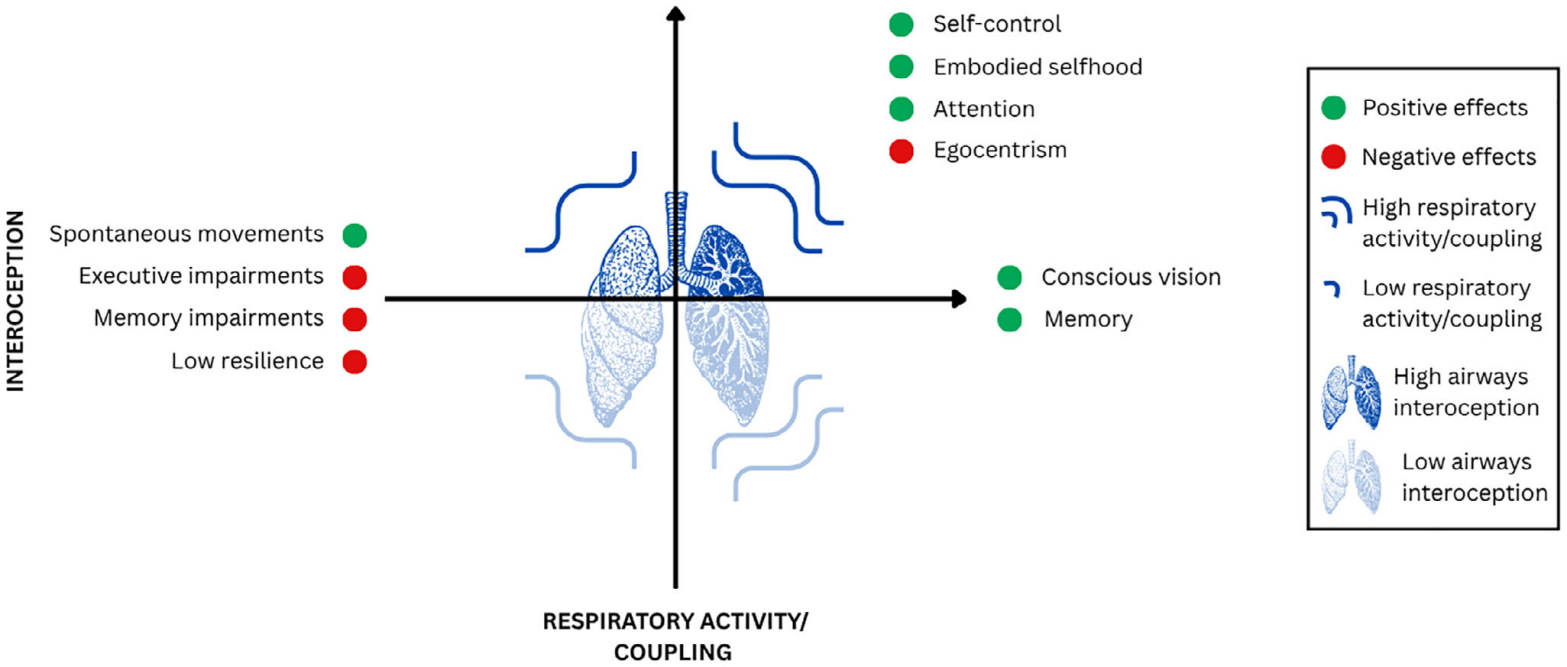
Impact of the respiratory system on higher cognitive functions, including the influence of respiratory signals coupled with brain, visual, or proprioceptive signals.

**FIGURE 4:**
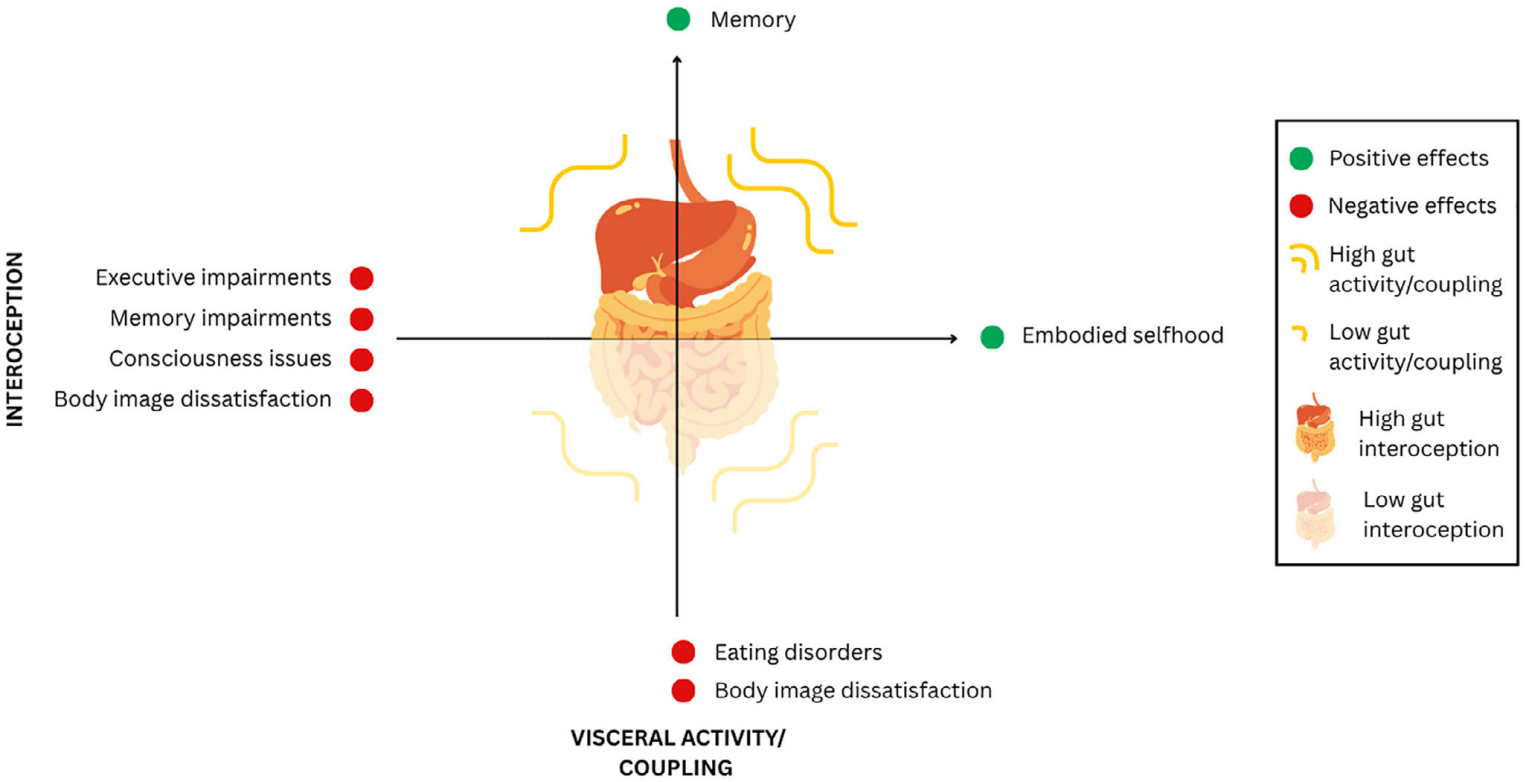
Impact of the gut, liver, and kidneys on higher cognitive functions, including the influence of gastric signals coupled with brain signals.

## 3 Respiratory Underpinnings of Cognition and Action

### 3.1 Breathing and Memory

If the importance of the heart in higher cognitive functions is becoming more and more acknowledged, other organs and physiological systems still lag behind. Among these, the respiratory apparatus is emerging as a surprising mediator of many mental operations. Like blood flow, also respiration displays a cyclical pattern of activity, alternating between inhalation and exhalation. A series of studies has recently highlighted the role of this pattern in memory encoding, consolidation, and retrieval. When a stimulus captures one's attention, it is likely that the attentional shift disrupts the respiratory rhythm and leads it to become aligned, that is, phase‐locked to the stimulus [110]. Specifically, showing words to be encoded at a fast rate induced participants to phase‐lock their breathing to the stimuli, and such a phase‐locking was stronger (deeper trough and higher peak) for words that were later remembered than for words that were ultimately forgotten. The larger the difference in breathing amplitude between remembered and forgotten words, the greater the difference of encoding‐related activity in the posterior midline region [110].

Breathing‐dependent activity in the amygdala and in the hippocampus shows different patterns at inhalation and exhalation, suggesting that the breathing phase can influence memory by modulating local field potentials [111]. A study with intracranial EEG revealed that nasal respiration entrains cortical activity in the amygdala, hippocampus, and piriform cortex, so that neurons in these regions oscillate in sync with the rhythm of nasal (but not oral) airflow [111]. Oscillatory power peaks around inhalation, and there is evidence that both memory encoding and retrieval are more accurate at inhalation than exhalation [111] (but see [112]). On the other hand, retrieval is less accurate when it takes place during the phase transition from exhalation to inhalation [113]. Nasal respiration enhances also the intermediate stage between encoding and retrieval, that is, consolidation, at least for olfactory memories [114, 115]. Finally, while a moderate level of experimentally induced dyspnoea does not impair memory performance, it does alter the event‐related potentials (ERPs) involved in recognition memory [116].

### 3.2 Breathing, Consciousness, and the Self

As long as we are alive, we breathe. The never‐ceasing cycle of inhalation and exhalation accompanies both rest and wakefulness. This respiratory rhythm is coupled to cerebral rhythms (respiration‐modulated brain oscillations) spanning all major frequency bands at rest [117]. Furthermore, in a near‐threshold spatial detection task, respiration was found to modulate both posterior alpha‐band oscillations (8–13 Hz) and the likelihood that the near‐threshold target was consciously detected or not [118]. According to the authors, given that alpha power is an established proxy of cortical excitability, and that its decrease prior to stimulus onset leads to increased stimulus detection, these results imply a three‐pronged relationship between breathing, neural excitability, and conscious perception. In another study, stimulating the olfactory epithelium with odorless air delivery increased slow (delta and theta) brain oscillations, reversed theta information flow from a wake‐like to a sleep‐like direction, and induced an altered state of consciousness, in which rational thinking and volitional control were reduced [119]. As a possible mechanism, nasal stimulation could trigger a burst mode activity in the medio‐dorsal thalamic nucleus (MDTN), which in turn would cause widespread delta‐theta oscillations [119].

The cyclical nature of respiratory signals and their impact on brain rhythms could also make them suitable for maintaining a stable sense of self across time [30]. Observing a virtual silhouette flash in sync with one's breathing cycles triggered a slight implicit shift of self‐location toward the silhouette [120] and illusory self‐identification with the silhouette [121]. Such effects were reduced when the silhouette flashed out of sync with breathing, and disappeared after experimentally induced dyspnoea, whereas mechanical ventilation, that is, machine‐delivered breaths, did not disrupt them [122, 123]. In the ‘embreathment’ bodily illusion [19, 124], we directly manipulated the representation of breathing on a virtual body, making an avatar breathe in phase with the participants’ breaths, in real time, or with an anti‐phase pattern. Participants were more likely to embody an avatar breathing in phase with them, as they reported higher ratings of ownership and agency toward the in‐phase virtual body. Breathing affected the sense of ownership almost as much as the appearance of the avatar. Respiration also impacted the sense of agency more than the appearance and the point of view from which the avatar was observed. Individuals with lower interoception experienced a stronger illusion, suggesting that their corporeal awareness is more amenable to manipulations. Overall, these results highlight the specific contribution of breathing to bodily self‐consciousness.

Respiration seems to be involved also in other forms of self‐related cognition. For example, inhalation makes it easier to discriminate one's voice from the voice of another person [125], possibly because inhalation drives changes in beta1 (12–16.5 Hz) EEG strength at rest, thereby facilitating attention to incoming stimuli [126]. Furthermore, a notable paper by Perl and colleagues [127] argues that when people touch their own face—an ubiquitous human behavior –they are in fact often sniffing their hands and thus smelling themselves. This peculiar form of nasal respiration might help establish or enhance people's sense of self, in addition to (or even in absence of) visual cues such as those provided by mirrors [127].

### 3.3 Breathing, Willed Action, and Decision Making

As self‐sniffing shows, a remarkable feature of breathing is that most of the time it is an entirely involuntary process, but healthy humans can control it voluntarily if needed. A fresh wave of experiments is revealing that breathing itself can impact the likelihood, timing, and quality of willed actions. Combining the classic Kornhuber and Libet tasks related to spontaneous self‐movements with respiratory measures, Park and coworkers [128] discovered that individuals are much more likely to spontaneously, consciously, willingly press a button during (late) exhalation than inhalation. Pressing a button is a voluntary motor command that likely competes with the motor command of respiration for neural and physical resources. Starting a voluntary action during the passive phase of the breathing cycle, that is, exhalation, can reduce such a conflict [128]. Of note, the link between respiratory phase and actions does not hold when an external trigger prompts participants to act, but is still present when they spontaneously imagine, rather than execute the movements [129]. On the other hand, inspiratory threshold loading slows locomotion control, as shown by a real and imagined timed up‐and‐go test 95. Again, this could be due to cortical competition between inspiratory loading and locomotor tasks [130].

Other studies sought to explore the link between breathing and self‐control. Per se, breath holding is not related to executive control [128]. However, individuals who correctly estimate, or even overestimate how much dyspnoea they will feel in an inspiratory breathing load task are also better at down‐regulating their craving, and show higher scores in a trait measure of self‐control [131, 132]. In an interoceptive inference framework [133, 134], this could be interpreted as suggesting that conservative predictions about future physiological states help the brain reduce surprise and achieve its long‐term homeostasis, avoiding failures of self‐control. Both interoception and self‐control recruit the anterior insula and pre‐supplementary motor area (preSMA), hinting at a common neural substrate [131, 132]. Furthermore, resistive load‐induced dyspnoea impairs response inhibition in a Stroop task both at the behavioral and neural level—yet another instance of possible competition for neural resources [135].

### 3.4 Breathing‐Related Processes and Other Forms of Higher‐Order Cognition

Recent studies are also unearthing a widespread role of breathing in a diverse set of other cognitive tasks. For example, self‐smelling may also play a role in social interactions. Sniffing one's right hand provides information about who one shook hands with, and occurs more often after within‐sex handshakes, whereas cross‐sex handshakes increase the likelihood that one sniffs one's left hand and thus samples one's own body odor [127]. Beyond olfaction, an experience of social exclusion increases feelings of dyspnoea [136], respiratory‐related evoked potentials [136], and negative affect linked to breath rate changes [137], at least in female samples. Further evidence about the impact of respiration on social cognition is provided by advanced yoga practitioners, who display reduced altruism as a function of yoga practice—arguably because intensely focusing on one's breathing, and on bodily feelings in general, induces a more self‐centered perspective [138]. Another form of mind‐body practice, diaphragmatic breathing training, increases sustained attention while reducing negative affect and cortisol level [139]. On the other hand, before and during a loaded breathing experience, people with low resilience display a greater insular and thalamic activation than average or highly resilient individuals, although they report to be less aware of interoceptive cues, suggesting a mismatch between attention and processing of respiratory signals may partly account for their lower ability to cope with stress, trauma, and adversity [140]. Respiratory variables change also as a function of cognitive load [141]. In a sample of airline pilot candidates performing a complex cognitive task simultaneously assessing perceptual speed, spatial orientation, and working memory, a stronger reactivity in respiration rate was correlated with better performance [142]. People are also more likely to start performing lexical, mathematical, and visuospatial cognitive tasks at inhalation than exhalation [126]. Inhaling at task onset predicts a better visuospatial performance, possibly because inhalation promotes attention by driving beta1 (12–16.5 Hz) EEG activity changes [126].

### 3.5 Respiratory Diseases and Higher‐Order Mental Functions

The recent COVID‐19 pandemic brought into the limelight the impact that impaired respiratory functions may have on cognitive functions. A large sample of people who recovered from COVID‐19 displayed remarkable deficits in a range of cognitive domains (attention, working memory, problem solving) scaling with the level of medical assistance patients received for COVID‐19 respiratory symptoms—in the worst cases, deficits exceeded those induced by stroke. Putative underlying causes of such effects include neurological changes, fatigue, and apathy [143]. Cognitive impairments have been reported also for chronic airway disorders such as chronic obstructive pulmonary disease (COPD) and chronic non‐obstructive bronchitis [144], although the specific impact of COPD on executive functions, memory, and attention over and above comorbidities is unclear [145, 146]. Patients suffering from another common respiratory condition, sleep‐disordered breathing, have a 26% increased risk of developing cognitive impairments and slightly poorer executive functions [147]. A possible explanation is that both COPD and sleep disorders may reduce the level of oxygen in the blood (hypoxemia) and in bodily tissues (hypoxia). For its part, low oxygen supply may lead to vasculopathy, neuronal loss, and disruptions of the blood‐brain barrier, ultimately resulting in reduced brain plasticity and cognitive problems [146, 147, 148, 149].

Lung transplant can improve respiratory conditions, but comes with an increased chance of cognitive impairment [150, 151], particularly in the executive functions and verbal memory domains [152]. These symptoms have been associated with prolonged allograft ischemic time [150] and delirium during hospitalization [151]. The issue may be compounded with pre‐operative comorbidities and post‐operative reduced grey matter volume in frontal, parietal, temporal, and insular cortices, as well as in cerebellar, putamen, and anterior cingulate areas [153]. However, post‐transplant pulmonary rehabilitation can improve cognitive functions, especially learning and memory [154].

Air pollution is another significant, if overlooked factor possibly mediating the relationship between breathing and cognition. Inhaled air pollutants may cause chronic neuroinflammation [155], while high levels of carbon dioxide (CO_2_) may lead to autonomic dysfunctions [156, 157]. In a brilliant study, Allen and colleagues [158] found that, compared to a working day spent in an environment simulating the indoor air quality of conventional buildings, higher‐order decision making scores improved by 61% when office clerks worked in a lab cubicle with a low rate of volatile organic compounds (VOC), and by 101% when low VOC levels were associated with high outdoor ventilation. Furthermore, independently of VOCs, increasing carbon dioxide (CO_2_) concentrations reduced cognitive performance in a linear fashion, up to 50%. A subsequent, 12‐month longitudinal study revealed that employees working in real‐world buildings with higher indoor concentrations of particulate matter (PM_2.5_) and lower ventilation had a poorer performance on the Stroop and addition‐subtraction tasks [159]. A schematic representation of the links between respiratory interoception and higher order functions is reported in Figure 3.

## 4 Gastric and Intestinal Underpinnings of Cognition and Action

### 4.1 Gut in Memory, Action, Self, and Social Cognition

If gut feelings have long entered into common parlance, it is only in the last few years that the stomach and the intestine emerged as promising enablers of higher cognitive functions, too. Water loading performance, a measure of gastric interoceptive accuracy, is correlated to logical memory percent retention, a proxy of hippocampal dependent learning and memory [160]. Indeed, the act of eating depends on memory for previous food intake and learned feeding routines, possibly through conditioned cephalic ghrelin secretion affecting hippocampal neurons [161]. Of note, the higher the amount of water ingested to reach satiation in the two‐stage water load test (WLT‐II), and the closer such amount was to perceived maximum stomach fullness, the more severe the self‐reported bulimic symptoms in a healthy female sample [20]. Gastric interoception is generally impaired in patients suffering from eating disorders [61], further underscoring the relationship between sensing one's stomach and feeding behavior. However, there is contrasting evidence on whether better gastric interoception, as assessed through the WLT‐II, is associated with a more positive body image [162] or not [163]. A study combining electroencephalography (EEG) with electrogastrography (EGG) found that the amplitude of spontaneous alpha rhythm fluctuations in the brain is coupled to the phase of gastric slow waves [164]. This prompted some researchers to assume that such a coupling is an implicit index of interoception, and examine whether it is related to body image concerns, finding that the weaker the gastric‐alpha coupling, the more negative the body image [165]. Women who are more dissatisfied with their body image also show poorer explicit interoception [124].

Beyond body image, now we have evidence that the physiological activity of the gut is correlated to corporeal awareness. In a recent experiment [166], we have used small, ingestible pills to wirelessly track the pressure, pH, and temperature of the stomach and the intestine (SmartPills) while healthy subjects underwent a bodily illusion in immersive virtual reality. According to our results, bodily self‐consciousness ratings tended to scale with gut parameters. When the gut was more active (as signaled by a more acidic stomach, a more basic intestine, or higher temperatures), participants had a better sense of self‐location and felt less disembodied. When the gut was less active, the reverse was generally true. Hence, we hypothesize that higher gut activity reinforces brain representations of one's own body, while weaker gut activity blurs them.

In addition to shedding light on the visceral roots of the bodily self, these data underscore the potential of minimally invasive, ingestible capsules to assess the links between gastro‐intestinal activity and mental states. Using the same technique, we discovered that the higher the acidity of the stomach, the stronger the disgust and fear people report after watching an emotion‐inducing video clip, while lower acidity correlates with greater happiness [167]. Another group found that mechanosensory feelings induced by a vibrating capsule elicit ‘gastric evoked potentials’ in parieto‐occipital areas [168].

In addition to chemical, electrical, and mechanical signals, the gut is also responsive to hormones and is home to a rich microbiota. An important peptide, oxytocin, may influence one's awareness of gastrointestinal and cardiac states through heart, gut, and ganglia receptors, and such awareness, in turn, may change one's social interactions: thus, oxytocin is emerging as a possible mediator of both eating and social behavior [169]. For its part, microbiota diversity has been linked to better executive functions and memory, and interventions changing the composition of gut microbiota through pre‐ or probiotics generally improve learning and memory [170, 171].

### 4.2 Gut and Other Forms of Higher‐Order Cognition

Combining EGG and functional magnetic resonance imaging (fMRI), Rebollo and colleagues [14] discovered that the slow rhythm of gastric contractions induced by the intrinsic electrical activity of the stomach fluctuates in delayed sync with a large network of brain areas at rest. The functional significance of this intriguing resting‐state network is not yet fully understood, and we speculate that it acts as a homeostatic regulator of food intake [172]. Although the EGG‐fMRI signal coupling occurs mostly in areas involved in resting‐state sensorimotor processing rather than cognition [173], future studies testing how such coupling changes when participants perform an interoceptive or cognitive task may contribute to clarify its role beyond rest and homeostasis. Finally, there is evidence linking altered gut signals with altered cognitive styles in some psychiatric conditions. For example, most children with autism spectrum disorder display remarkable gastro‐intestinal issues—including gastroesophageal reflux, constipation, diarrhea, vomiting, intestinal infections, increased intestinal permeability, and altered microbiota [174]—and such issues are particularly related to repetitive behavior [175]. Furthermore, repetitive negative thinking in patients with major depressive disorder correlates with weaker activity in the right medial temporal lobe, including the hippocampus and amygdala, during a gastric interoceptive attention task [176]. Abnormal changes in microbiota feature also in neurological disorders like Parkinson's disease [177] or multiple sclerosis [178, 179], and are linked to cognitive deficits observed in these conditions [179, 180]. A schematic representation of the links between gastrointestinal interoception and higher order functions is reported in Figure 4.

## 5 Contributions of Other Visceral Organs to Cognition and Emotion

### 5.1 Liver

Thanks to studies on organ transplants and organ disfunctions, it is becoming more and more apparent that there are surprising connections between cognitive functions and the activity of visceral organs other than the heart, the lungs, and the gut. As a case in point, up to 50% of liver transplant recipients show postoperative cognitive dysfunction (POCD), mainly because surgery triggers an increase of neuroinflammatory activity [181]. POCD affects higher‐order functions such as short‐term memory, long‐term memory, and consciousness [181, 182]. Global and domain‐specific cognitive impairment often persists after more than 6 months from the transplant [183]. POCD can be present even in living liver transplant donors, who show reduced executive functions, as assessed by trail making and Stroop tasks 1 week after the transplant [184].

While liver transplant is often performed to tackle alcohol‐related cirrhosis, liver problems may occur also when alcohol consumption does not exceed the average, as shown by non‐alcoholic fatty liver disease (NAFLD). According to observational studies, NAFLD patients tend to have poor general cognition, mental speed, abstraction, and mental flexibility, possibly on account of a general metabolic syndrome that in turn affects brain cells and thus impairs cognition [185] (but see [186]). Another related issue, liver fibrosis, was associated with worse performance in tests of immediate recall [187] and executive function [188] in very large samples of patients, as well as with reduced hippocampal and total brain volume [188], implying that a ‘brain‐liver axis’ can impact cognition [189].

### 5.2 Kidneys

Chronic kidney disease and end‐stage renal disease can impair cognition by increasing the chance of cerebral atrophy and other cerebrovascular disorders, an effect compounded with fluid and osmotic shifts induced by dialysis [190] and possibly with uremic toxins [191]. Kidney transplantation can mitigate such disease‐induced cognitive deficits [192, 193, 194], improving memory and executive function [195]. However, it is unclear if such benefits hold only for relatively young patients or across the age spectrum [190]. Moreover, between a third and a half of kidney transplant recipients keep showing cognitive deficits in executive function and other domains [196, 197], and subjective clinical evaluations often fail to acknowledge such deficits [198].

### 5.3 Bladder

While the liver and the kidneys do not elicit conscious interoceptive feelings apart from referred pain, there are specific sensations associated with the bladder, including perceived fullness and urge to void. Such conscious perceptions are altered in overactive bladder syndrome (OAB). Compared to healthy controls, OAB patients report an increased urge and feel their bladder is full even when it is not, as shown by lower voided volumes, but they also display higher self‐awareness scores [199], suggesting their bladder cues are not properly integrated in their bodily self. Finally, mild cognitive impairment seems more prevalent in bladder cancer patients than in the general population of the same age class [200].

### 5.4 Ovarian Hormones and Free Nerve Endings

In women, a further visceral pathway to cognition is represented by ovarian hormones. In particular, estradiol impacts on the structure and function of the hippocampus and prefrontal cortex during memory tasks [201] (cf. [202]). Episodic and verbal working memory circuits are affected by menopause, and such effects correlate with estradiol decline [203, 204], while hormone therapy at early post‐menopause enhances prefrontal activity related to cognitive control [205]. Our group also found that the closer women get to their monthly premenstrual phase, the greater their body image concerns [124].

In both sexes, another source of interoceptive signals that can matter to higher cognition is represented by free nerve endings detecting changes in affective touch and temperature. Slow, affective touch, detected by specialized C‐tactile receptors, induces both an increased sensation of pleasantness and stronger embodiment in the rubber hand illusion, suggesting that one feels a hand belongs to oneself also thanks to affective tactile cues [91, 206], although affective touch does not influence the embodiment of the whole body [207]. Finally, an assessment of cognitive functions during a heatwave found that excessive temperature was associated with slower cognitive speed and worse working memory in young adults [208], suggesting that also thermal sensations may play a role in cognition [209]. Indeed, body temperature seems to correlate with the sense of body ownership [210, 211, 212] (cf. [213]), possibly owing to shared brain areas as the insula [211], while people are more likely to feel the need for social connections when it is relatively cold, and perceive others as more sociable when it is relatively warm [214].

## 6 Toward a Deep Embodiment Paradigm

Since the dawn of modern neuroscience, the brain has been increasingly regarded as the sole seat of thought, taking the role that was previously fulfilled by the heart and by breathing. Here, we showed that many other organs are involved in the higher forms of thinking that are the hallmark of our species, and if such organs are not healthy, cognitive impairment follows. This evidence can be hardly reconciled with what we may term “brain reductionism”—the claim that “a person's mental activities are entirely due to the behavior of nerve cells, glial cells, and the atoms, ions, and molecules that make them up,” as Francis Crick famously hypothesized [215] (for a discussion, see [216]). Alongside neural mechanisms, it is rather plausible that any truly integrative and holistic vision of the biological substrates that sustain mental processes must also encompass structures and processes that are not primarily neural.

At the same time, it would not be enough to adopt a classical embodied approach, because standard embodiment theories focus on the outer, rather than the inner body. Instead of this “shallow embodiment,” we should adopt a “deep embodiment” standpoint, acknowledging that the activity of visceral organs is implicated in memory, willed action, consciousness, and social cognition. Across these domains, the influence of heartbeats and breathing is strongly modulated by their cyclical nature. At systole, the increased inflow of visceral inputs anchors cognition to afferent bodily signals and ultimately to the self: social behavior becomes more egocentric [95] and ingroup‐oriented [93], social pain is reduced [98] and actions seem to be more easily attributed to oneself [67] than at diastole. Likewise, during inhalation there is a higher chance of initiating self‐controlled cognitive tasks [126] and discriminating between self and non‐self [125] than at exhalation. Hence, we propose to distinguish between a more ‘self‐centered mode of thinking’ at systole or inhalation, and a ‘nonself‐centered mode of thinking’ at diastole or exhalation. Self‐centered cognition is further promoted when interoception is high (see e.g. [65, 70, 71, 72, 95, 97]), while low interoception has the opposite effect [19, 76] (Figure 5, right). This cyclical switch between self‐centered and a nonself‐centered thinking may help agents reach a ‘cognitive homeostasis’, that is, an optimal balance between processing internal signals and gathering external information. A potential challenge for our framework is the finding that spontaneous, voluntary actions are more likely at diastole [75] or at exhalation [128] than at systole or at inhalation. However, by definition such actions, while firmly in control of the self, imply the willingness to engage with the external environment without a specific goal, thus fitting quite well in a nonself‐centered mode. Cardiac and breathing cycles are also reflected in their neural counterparts, like heartbeat‐evoked responses (HERs) and respiration‐modulated brain oscillations. Oscillations induced by heartbeats and breaths can help cross‐regional communication in the brain [13, 217], linking interoceptive signals to cortical firing. While interoceptive and cognitive areas sometimes overlap, as in the case of hippocampus [160], it is currently difficult to pinpoint cerebral areas that are both necessary and sufficient to integrate interoceptive and cognitive information. For example, the insula may be a significant contributor to both interoception and cognition [218], but it is not included in the gastric resting‐state network [14] and insular damage does not suppress cardiac interoception [219]. Nevertheless, the fact that cardiac and respiratory rhythms can set the pace of brain activity and thus shape cognitive functions like consciousness [43, 45] or memory [126] reveals the actual existence of what we called a *first‐order pathway of visceral cognition* (section 1), that is, a direct influence of visceral physiology on cognitive operations. We have evidence also for our proposed *second‐order pathway of visceral cognition*, that is, an organ supporting another organ that affects cognition: for example, kidneys improve the performance of the cardiocirculatory system, which in turn shapes cognitive performance. Other data are consistent with our *third‐order pathway of visceral cognition*, in which the viscera indirectly steer cognition by influencing the general state of brain and body: the respiratory system carries atmospheric gases and pollutants to the rest of the organism [158, 159], while thermoreceptors signal adverse heat conditions to the central nervous system [209]. These three pathways can be thought of as comprehensive physiological mechanisms orchestrating the interplay between viscera and cognition at different timescales and in different anatomical regions (Figure 5, left).

**FIGURE 5:**
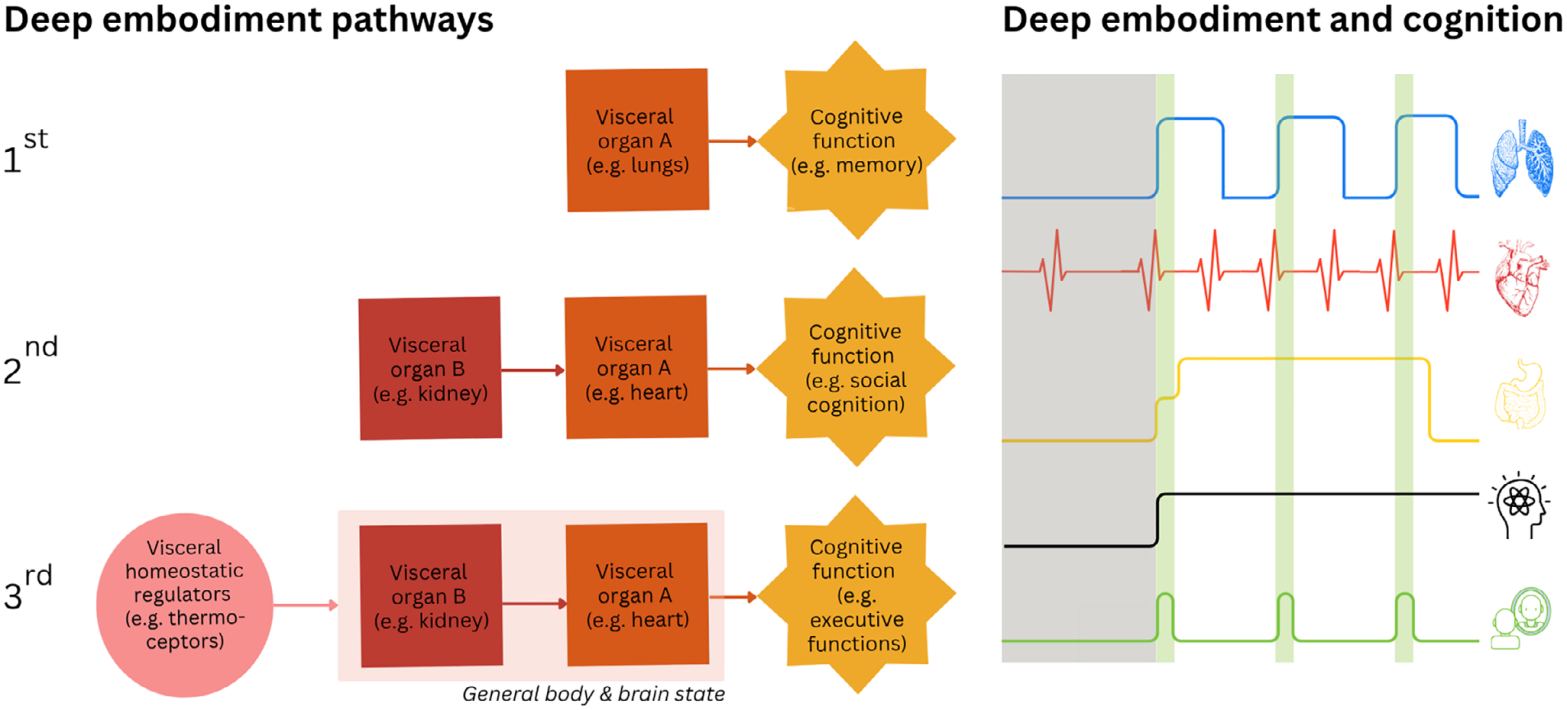
Left: the three pathways of deep embodiment (first‐order, second‐order, and third‐order influence of visceral organs on higher cognition). Right: different frequencies of respiratory (blue), cardiac (red) and gastrointestinal (yellow) signals impact on cognitive functions, so that states of high visceral activity and high interoception generally promote cognition (black), particularly when interoception is high. Cardiac systole and inspiration likely boost self‐focus (green).

The gut seems to be mostly linked to self‐ and food‐related cognition, while the liver, the kidneys, the bladder, and the ovaries tend to be associated with memory and executive functions. However, the narrower cognitive scope of these organs may simply reflect the fact that there is still a limited number of studies on them, and memory and executive functions are easier to test in clinical settings. Probing other cognitive domains and sampling also non‐clinical populations will help understand if these viscera play a wider role in thought processes.

Regrettably, there is a clear gap between studies that assess explicit interoception through questionnaires or objective tests of performance, and studies that measure implicit interoception through electrophysiological recordings or brain imaging. Both layers of interoception should be gauged within the same study, but this is rarely the case. While a number of experiments report cognitive effects of implicit interoception that are independent from explicit interoception, like the effects of HERs on self‐related cognition, there is evidence correlating the amplitude of HEPs with performance in the HCT [220].

Standard tests of interoception, like the HCT, are primarily focused on cardiac feelings and have been recently subject to methodological criticism [221, 222, 223]. To foster further progress in the field, we need better methods to record the conscious perception of cardiac signals [224] as well as new techniques to probe non‐cardiac interoception [19] and physiology, including wearable sensors [225], high‐resolution electrogastrography [226], and ingestible pills [166, 167]. Cardiac and respiratory interoception seem to be dissociated from each other [227], but cardiac and gastric interoception are correlated [228]. Hence, future research should devote more effort to study multi‐organ visceral cognition. To test whether multiple organs are connected in a single causal pathway, one should record all their physiological signals at once while increasing or inhibiting the activity of one or few organs at a time, capitalizing on behavioral manipulations (e.g. exercise, fasting), pharmacological protocols (e.g. β‐adrenergic agonists vs. antagonists) or electromagnetic stimulation (e.g. excitatory vs. inhibitory effects of vagus stimulation).

Taking into account the three different orders of visceral influence on higher cognition can also improve the design of medical digital twins [229], that is, artificial intelligence (AI) models simulating the biology of the human body. For example, one could use digital twins of the human heart [230] to simulate and predict how people think and behave in a given ‘state of the heart’. The same applies to digital twins of the lung [231] or other organs. Given the links between visceral activity and nervous activity, incorporating visceral information can also help refining in silico models of the brain [232] and particularly mental health digital twins [233], with applications in precision psychiatry and psychotherapy [234].

If science is about finding causes, it must be acknowledged that the science of interoceptive influences on higher‐order cognitive functions is still in its infancy. More experiments adopting a causal approach are needed to establish if higher cognition is truly dependent on the viscera rather than simply accompanied by co‐occurring physiological activations. However, the evidence accrued so far has already changed our understanding of the bodily underpinnings of memory, action, consciousness, and social interactions. Our higher‐order cognition is not merely embodied, but deeply embodied—it pulses with our heartbeats, it pangs with our gut, and it flows with our breath.

## Author Contributions

Conceptualization: AM, SMA; Visualization: AM; Supervision: SMA; Writing – original draft: AM; Writing: review & editing: AM, SMA; Funding: SMA.

## Funding

ERC Advanced Grant eHONESTY (789058), HORIZON‐ERC‐POC DeepDive (101138232)

## Conflicts of Interest

The authors declare no conflicts of interests.
